# An Alternative Pharmacological Therapy for Obesity Through Modulation of Vagus Nerve Synaptic Receptors

**DOI:** 10.3390/jcm15207914

**Published:** 2026-10-14

**Authors:** Claudio Blasi

**Affiliations:** Centro Diabete, Ospedale Sandro Pertini, 00157 Rome, Italy; diabcb@gmail.com

**Keywords:** treatment of obesity, vagus nerve, NMDA receptors, GluN2B (NR2B) subunit, bariatric surgery, food addiction

## Abstract

Obesity is a rapidly spreading disease globally. It causes serious complications, including type 2 diabetes, hypertension, dyslipidemia, fatty liver disease, and other serious cardiovascular consequences. Weight loss, the primary driver of the healing process, is extremely difficult to achieve due to the body’s strong resistance, a legacy of an evolutionary process that protected the human species from extinction due to nutritional deficiency. The few drugs currently available and recognized as effective, however, are not free from side effects and, more importantly, do not guarantee a permanent cure, but only a temporary suspension of symptoms during administration. Bariatric surgery, however, has proven highly effective. The ideal goal would be to find an equally effective pharmacological solution that reproduces the same mechanism. Currently, only hypotheses exist. Many clues point to a modified function of the ascending vagal afferent fibers that connect the gastro-intestinal tract to the brain. In this narrative, hypothesis-driven review, it is hypothesized that pharmacological modulation of glutamatergic (NMDA) receptors at the synapses between vagal afferent fibers and their central targets may represent a novel therapeutic strategy for obesity. This approach would target the putative altered mechanism present in obesity, with the hypothetical potential to promote sustained weight loss or durable disease modification, pending experimental and clinical confirmation. There are already some encouraging signs in this direction.

## 1. Introduction

Obesity is a major cardiovascular risk factor with a rapidly increasing prevalence in the global population. According to World Health Organization data, the global prevalence of obesity has doubled over the past 30 years [1]. The percentage of overweight individuals reaches 45%, while approximately 15% are affected by obesity, representing millions of people. This prevalence of excess weight is projected to rise among both the adult and youth populations, constituting a major global health issue [1].

The existing therapeutic approaches for this condition are limited by the body’s strong resistance to weight loss. This resistance is mainly attributable to two brain regions that regulate energy homeostasis: the hypothalamus and the reward circuit. In the case of hypothalamus, the “set-point” theory posits that weight loss across a predetermined interval is hindered by evolutionary advances aimed at protecting the survival of the species by preventing the loss of adipose tissue (as energy reserve) in the presence of food shortage [2]. In the case of reward circuit, as described in detail later, resistance stems from dependence on palatable, high-calorie food (CF), which limits the individual’s ability to adhere to a specific diet. For these reasons, obesity treatment based solely on lifestyle changes (e.g., low-calorie diet and increased physical activity) achieves poor results.

Pharmacological approaches also offer limited options. Among the existing pharmacological agents, GLP-1 analogs, a hormone produced by enteroendocrine L-cells, have led to significant weight loss and regression of diabetes and cardiovascular complications. However, these agents are expensive and not always available. Moreover, they can cause adverse reactions affecting the digestive system, including abdominal pain, nausea and vomiting, constipation or diarrhea, and changes in the cells of the exocrine pancreas with the risk of pancreatitis [3,4]. Furthermore, their effects may not be sustained after treatment discontinuation: weight regain has been reported in many patients, together with a loss of cardiometabolic benefits [5]. It has been suggested that these agents may not fully address the functional alterations underlying obesity, and that their benefits may depend on continued treatment [6].

Since obesity is a chronic relapsing disease, an appropriate pharmacological approach is necessary to ensure lasting remission [7]. Is it possible to undertake an alternative therapeutic path? This narrative, hypothesis-driven review aims to point out to experimental research a possible alternative to pharmacological obesity treatment, one that utilizes substances acting on receptors for the vagal neurotransmitter glutamate.

The selection of literature constitutes an impartial overview of the most recent work on the specific topics addressed. It was identified through targeted searches of Medline/PubMed and was selected non-systematically to illustrate and support the proposed hypothesis.

## 2. The Glimmer of Hope Provided by Bariatric Surgery

Bariatric surgery (BS), which was initially performed for predominantly aesthetic reasons, has proven highly effective in achieving substantial and sustained weight loss and regression of diabetes as well as other complications [8]. Sleeve gastrectomy and gastroduodenal bypass (Roux-en-Y gastric bypass [RYGB]) are two options for BS. In sleeve gastrectomy, the most commonly performed procedure, typically performed laparoscopically, the stomach is reduced to a small sleeve-shaped tube. In contrast, gastroduodenal bypass involves creating a small gastric pouch and connecting it to the small intestine about 20 cm from the cardia, thereby excluding the rest of the stomach and duodenum from contact with food.

However, the mechanisms underlying the positive clinical results of BS remain unclear to date [8]. Identifying these mechanisms and reproducing them with pharmacological agents could avoid the drawbacks of surgery, including costs, complications, and limited availability of specialized centers, potentially creating an important new therapeutic option.

In this regard, numerous studies have suggested that metabolic surgery modifies vagal signaling from the gastrointestinal (GI) tract to the central nervous system (CNS) by altering the anatomical integrity and functional properties of the gastrointestinal tract [9,10,11,12]. In particular, a detailed examination of surgery-induced changes can yield important insights covering various aspects, including restoring the normal body weight set-point, elimination of the weight maintenance response, activity of the gastric pouch formed by the surgery, isolation of the duodenal wall from food, changing eating habits (i.e., the choice of lower-calorie foods), modification of vagal control over pancreatic insulin secretion and over hepatic glucose production, and secretion of incretins. These effects may be attributable to the modification of the activity of the so-called vagal “gut-brain axis” (For further details, please see Ref. [13]).

## 3. Composition of the Gut–Brain Axis

The muscular layers and the mucosa of the GI tract are innervated by a nervous network (known as the “intestinal brain” or “second brain”) that is activated by the passage of the food bolus. For this network, the food bolus acts as both a mechanical stimulus, causing wall dilation, and a chemical stimulus, inducing hormone production by the enteroendocrine cells in the epithelium [14,15]. These stimuli activate the vagal nerve endings present in the wall, and the vagal afferent fibers (VAFs), which consist of pseudounipolar neurons, transmit signals to the cells of the nucleus tractus solitarius (NTS) in the brainstem through glutamatergic synapses (see below) [12,16].

Subsequently, other vagal fibers form glutamatergic synapses with neurons in various brain sectors involved in energy homeostasis [12,17]. VAFs also connect with the sites of the “reward” circuit [17], which plays an important role in food addiction [17].

Efferent vagal fibers also originate from the NTS, connecting it to splanchnic organs such as the liver and the endocrine pancreas [16]. Thus, the vagus nerve constitutes a crucial bidirectional communication pathway for mediating metabolic control [12].

Control of afferent vagal function is also exerted by endogenous opioid peptides, which inhibit the transmission of signals within the NTS mediated by the neurotransmitter glutamate. In this regard, enkephalin, an important opioid agonist, acts as a neuromodulator, regulating the transmission of impulses arising from vagal afferent fibers and thereby influencing the activity of the gut–brain axis, transforming it from static to adaptable [18].

## 4. How Does Vagal Signal Transmission Occur?

VAFs connect to neurons in the NTS and brain centers via presynaptic secretion of the neurotransmitter glutamate, which activates receptors on the postsynaptic neurons [19,20].

The most important receptor in this process is *N*-methyl-d-aspartate (NMDA), a transmembrane protein that opens in response to the neurotransmitter, allowing the transfer of the nerve impulse. It is composed of four distinct protein subunits (or monomers): GluN1, GluN2A–D, GluN3A–B [21,22].

Pharmacological regulation of glutamatergic transmission, the predominant mode of transmission in the CNS, has demonstrated considerable therapeutic potential across various medical fields. However, it may also be associated with adverse reactions, including impaired cardiovascular function, impaired motility, hallucinations, and delusions [23]. In this regard, low-affinity receptor subunit antagonists, which inhibit the action of glutamate by blocking glutamate-binding sites without completely stopping neurotransmission, are more advantageous with a more acceptable adverse effect profile [24].

The GluN2B (formerly NR2B) subunit is the most suitable antagonist for this purpose. By acting on the sites for allosteric ligands (molecules that bind to a region of the receptor that is different from the principal active site), it can achieve an effective pharmacological action with limited adverse reactions [25,26] (See below).

## 5. Obesity as a Form of Addiction

Treatment of obesity should take into account its pathophysiological characteristics, including the addiction of CF. Addiction refers to a tendency toward compulsive behaviors that include reduced control over use, persistent cravings, or unsuccessful efforts to reduce consumption despite awareness of its harmfulness [27,28,29].

CF addiction is, in many aspects, similar to other forms of addiction such as drug addiction [30]. The origin of excessive food intake not justified by homeostatic survival needs has been identified in the brain’s mesolimbic system, known as the “dopaminergic circuit” or reward circuit.

In this circuit, rewarding experiences trigger the secretion of the neurotransmitter dopamine and its diffusion into adjacent regions. However, improper intake of a CF-based diet reduces the sensitivity of D2 dopamine receptors, making the brain less able to experience pleasure from natural stimuli. Moreover, the so-called “neuronal plasticity” is activated in the mesolimbic dopaminergic circuit [31]. Plasticity changes also occur in VAF targets [32].

Neuronal plasticity refers to the brain’s ability to self-regulate, maintaining stable electrical activity without losing control of intersynaptic transmission. An important aspect of neuronal plasticity is the ability of synapses to strengthen or weaken in response to functional stimuli [33]. However, prolonged exposure to chronic environmental stimuli (such as alcohol and CF abuse) increases glutamatergic activity, which leads to the activation of NMDA receptors (NMDARs upregulation) [34]. Morphological alterations in neurons, such as an increase in size and dendritic arborization, are associated with these changes [35].

Studies of food addiction may take advantage of the similarity of this condition to alcohol addiction. Indeed, alcohol abuse and obesity involve overlapping neurocircuitry within the brain’s reward and appetite pathways, and they are also influenced by similar neuroendocrine and neuroimmune signaling pathways [36]. Positron emission tomography (PET) and functional magnetic resonance imaging (fMRI) studies using visual, olfactory, and gustatory stimuli have demonstrated that food and alcohol stimuli activate overlapping neural networks [36]. Thus, the receptor alterations that contribute to the development of ethanol dependence, as well as to the acute and delayed withdrawal symptoms, can plausibly be the same as those involved in CF dependence [36,37]. Therefore, they may respond similarly to treatments targeting their common pathophysiological mechanisms [36].

## 6. How Can Therapeutic Interventions Be Implemented?

Neural plasticity in feeding circuits is a key component for controlling energy balance [38]. When this process is altered, the organism’s energy homeostasis may be compromised [39].

In obesity, brain sectors involved in weight control and the reward system may lose sensitivity to VAF inputs due to synaptic plasticity [30]. Therefore, it is hypothesized that modulation of intersynaptic transmission may be a valid pharmacological approach [38].

Given the important pathophysiological role of CF addiction in these patients, one of the goals of obesity therapy is to correct this dysfunctional aspect. The structural changes associated with addiction may be inhibited by substances that block the activity of NMDARs [40], especially the GluN2B subunit [26]. Increased expression of this subunit has been identified in the development of alcohol dependence [37,41,42]. Thus, the prospect of an effective pharmacological intervention targeting GluN2B shows promise for both alcoholism and hyperphagia [37,43,44]. Some positive findings have been obtained in this regard. For example, during ethanol withdrawal, treatment with ifenprodil (a GluN2B-selective antagonist) [45,46] has been shown to significantly reduce the expression of symptoms [26].

Non-selective effective NMDAR channel blockers with a broader pharmacodynamic profile are ketamine and its optical isomer esketamine [47]. Their clinical effects represent indirect evidence supporting the efficacy of NMDAR blockade in general. Short-term ketamine treatment may lead to improvements in neurological functions that last longer than its pharmacokinetic half-life [21,47]. Ketamine has been proven to be a highly effective antidepressant through a novel mechanism, unlike drugs that increase serotonin availability: the induction of persistent functional changes in the brain involving neuroplasticity [21]. Ketamine’s mechanism of action may be linked to homeostatic synaptic plasticity processes triggered by the suppression of NMDA receptor-mediated glutamatergic neurotransmission [48].

Indeed, impaired neural plasticity and dysfunction of NMDARs caused by an altered glutamatergic signaling has been hypothesized to be a cause of depression resistant to conventional treatments [49,50]. Thus, inhibition of the receptor and the restoration of normal neurotransmission would explain the therapeutic effect [51].

Interestingly, treatment of major depression is more effective in the presence of obesity, suggesting some pathophysiological link between the two conditions [52]. Ketamine, in particular, unlike other classic antidepressants, shows favorable responses in patients with high BMI, particularly in those with abdominal obesity [53,54], typical of metabolic syndrome: this is of further interest.

Finally, neurosteroids represent another class of NMDAR blockers that can lead to the development of drugs with interesting therapeutic prospects [55]. In particular, at micromolar concentrations, the neuroactive steroid pregnenolone sulfate acts as an allosteric modulator for a variety of glutamate receptors [56] and could be tested in obesity.

## 7. Hypotheses for the Therapeutic Effects of BS

Understanding the therapeutic mechanisms of BS is crucial for developing future non-surgical treatments for obesity [57]. As described previously, a strong body of evidence suggests that BS alters how the gut communicates with the brain via the vagal afferent system [8,13].

According to this hypothesis, BS “resets” the neurological system that presides over metabolic homeostasis and control of nutrition to (A) bring the hypothalamic set-point (and therefore the reference weight) back to a normal level [57] and (B) restore correct sensitivity to nutrients by reconstituting the dopaminergic signaling activity in the mesolimbic area (unlike what happens with weight loss induced by diet alone) [9]. Thus, indirect evidence suggests that the taste for non-high-calorie foods would also be restored [44].

These effects may arise from a rapid form of homeostatic plasticity, a process that stabilizes synaptic activity, involving the reorganization and reprogramming of NMDARs [21,47]; hypothetically, the process proposed for ketamine could also operate in obesity, through suppression of NMDAR-mediated glutamatergic neurotransmission.

## 8. How to Use NMDAR Reprogramming in Obesity?

The increased interest in the therapeutic potential of NMDA blockers is also linked to the clinical success of ketamine [21]. It is hypothesized that such low-affinity, rapidly dissociating blockers whose mechanism of action could be linked to homeostatic synaptic plasticity processes activated after suppression of NMDA-mediated glutamatergic neurotransmission, will probably be considered in further clinical settings [47,58], like, for example, obesity.

Thus, the improvement in patients’ condition would most likely occur through neural plasticity, which counteracts synaptic dysfunction [47,59]. The molecular and ultrastructural mechanisms underlying this restoration are unknown.

Accordingly, new NMDAR modulators have entered clinical development [25]. In particular, esketamine, an optical isomer (i.e., a mirror molecule) of ketamine, acts with the precision of a scalpel, reversing impaired neural plasticity and restoring correct synaptic function [60,61].

Research on NMDAR modulators is ongoing. GluN2B-selective antagonists such as traxoprodil have been tested in clinical studies in depression, and ifenprodil has been investigated in preclinical models of alcohol dependence, while ketamine and esketamine are already in clinical use. No studies have so far tested these compounds specifically in obesity.

However, the choice of pharmacological agents and the optimization of NMDAR modulator use must be carefully considered to fully exploit their therapeutic potential [21]. This has yet to be experimentally demonstrated.

## 9. What Are the Future Prospects for This Path?

It has been described above how set-point weight deviations and CF dependence can be linked to altered homeostatic plasticity (cf. [62]) and highlighted the potential therapeutic role of NMDAR modulators [51,63].

GluN2B is among the GluN2 subunits expressed in the adult brain [64] and presents various recognition sites for allosteric ligands, making it a particularly rich pharmacological target [25]. Allosteric modulators are considered reference molecules for the development of future drugs indicated for multiple neurological pathologies [65].

Future research, in combination with new technologies, will enable better characterization of the GluN2B subunit and provide a better understanding of the functional consequences of its alterations [25,26]. This would allow the development of next-generation agents selective for this subunit, facilitating increasingly precise targeting suitable for clinical development [21]. Furthermore, NMDAR modulators linking their mechanism of action to homeostatic synaptic plasticity processes activated after suppression of NMDA-mediated glutamatergic neurotransmission could be tested in appropriately designed preclinical and clinical studies in obesity. Evidence for this is indirect and supports only the biological plausibility of the hypothesis.

However, the translation of experimental studies into clinical-therapeutic applications is hindered by the complexity of the functions of the receptor and the subunits involved in different brain processes [66].

## 10. Conclusions

**Figure 1:**
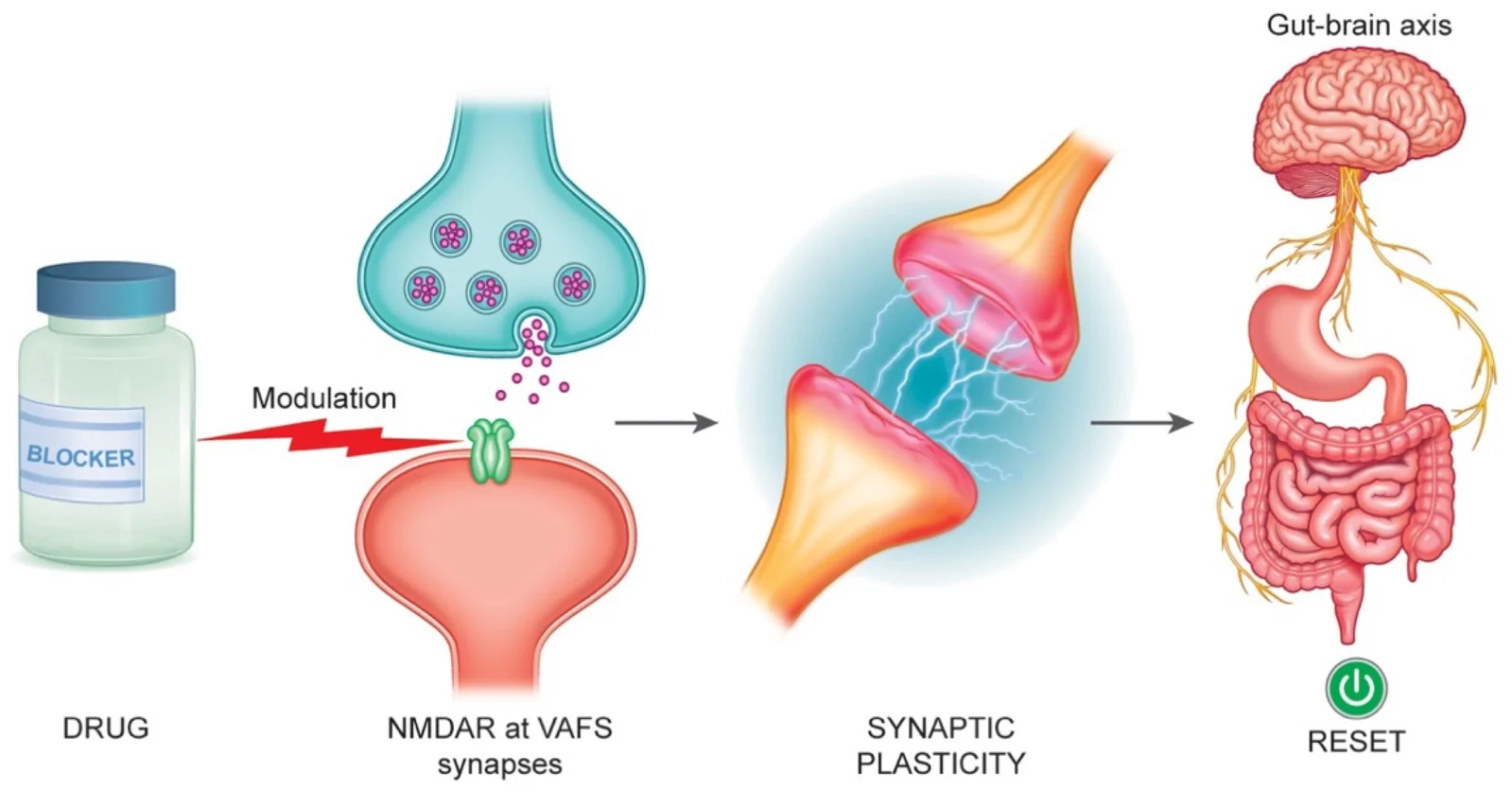
Hypothetical model of an alternative pharmacological approach to obesity: modulation of activity of synaptic NMDA receptor on vagal afferent terminals to achieve a reset of the GI-brain axis through synaptic plasticity.

Although no clinical evidence has currently established that pharmacological modulation of the NMDAR at VAF synapses could play a major role, it represents a potential therapeutic alternative for obesity [66]. This innovative pharmacological approach can enable functional restoration of the gut–brain axis, yielding sustained improvements in synaptic function [43]. It can be hypothesized that pharmacologically induced synaptic plasticity may produce effects persisting beyond the period of direct drug exposure (Figure 1). However, the possibility that such effects translate into sustained weight loss requires experimental and clinical investigations, which will hopefully be undertaken.

NMDAR modulation is a candidate target worthy of further investigation as a therapeutic strategy for obesity. Future research can be expected to confirm this receptor as the primary regulator of vagal synaptic transmission and highlight its amenability to effective therapeutic intervention in obesity.

## 11. Limitations

This review has several limitations: the lack of direct clinical evidence; the reliance on indirect and preclinical evidence; the uncertain anatomical specificity; and the uncertainty as to whether NMDAR-related plasticity is causal or associative; the literature identified through targeted searches. Experimental and clinical confirmation is therefore needed before NMDAR modulation can be considered as a future therapeutic option for obesity.

## Data Availability

No new data were created or analyzed in this study. Data sharing is not applicable to this article.

## References

[1] World Health Organization Obesity and overweight [Internet].

[2] Garvey W.T. (2022). Is obesity or adiposity-based chronic disease curable: The set point theory, the environment, and second-generation medications. Endocr. Pract..

[3] Sodhi M., Rezaeianzadeh R., Kezouh A., Etminan M. (2023). Risk of gastrointestinal adverse events associated with glucagon-likepeptide-1 receptor agonists for weight loss. JAMA.

[4] Cohen D. (2013). Reports of pancreatitis are 20-30 times more likely with GLP-1 drugs, analysis finds. BMJ.

[5] West S., Scragg J., Aveyard P., Oke J.L., Willis L., Haffner S.J.P., Knight H., Wang D., Morrow S., Heath L. (2026). Weight regain after cessation of medication for weight management: Systematic review and meta-analysis. BMJ.

[6] Perry Wilson F. (2025). What Comes Back When Stopping GLP-1s?. Medscape Comment.

[7] Forner P., Hocking S. (2025). Pharmacotherapy for the management of overweight and obesity. Aust. J. Gen. Pract..

[8] Cook T.M., Bethea M.M., Sandoval D.A. (2025). The role of the gut-brain axis in bariatric surgery. Curr. Opin. Neurobiol..

[9] Richards P., Thornberry N.A., Pinto S. (2021). The gut-brain axis: Identifying new therapeutic approaches for type 2 diabetes, obesity, and related disorders. Mol. Metab..

[10] Berthoud H.R., Shin A.C., Zheng H. (2011). Obesity surgery and gut-brain communication. Physiol. Behav..

[11] Ballsmider L.A., Vaughn A.C., David M., Hajnal A., Di Lorenzo P.M., Czaja K. (2015). Sleeve gastrectomy and Roux-en-Y gastric bypass alter the gut-brain communication. Neural Plast..

[12] Borgmann D., Fenselau H. (2024). Vagal pathways for systemic regulation of glucose metabolism. Semin Cell Dev. Biol..

[13] Blasi C. (2025). Mechanisms of metabolic surgery effectiveness in obesity and type 2 diabetes: A puzzle with some known pieces. Int. J. Obes..

[14] Powley T.L., Spaulding R.A., Haglof S.A. (2011). Vagal afferent innervation of the proximal gastrointestinal tract mucosa: Chemoreceptor and mechanoreceptor architecture. J. Comp. Neurol..

[15] Berthoud H.R., Kressel M., Raybould H.E., Neuhuber W.L. (1995). Vagal sensors in the rat duodenal mucosa: Distribution and structure as revealed by in vivo DiI-tracing. Anat. Embryol..

[16] Watts A.G., Kanoski S.E., Sanche G., Langhans W. (2022). The physiological control of eating:Signals, neurons, and networks. Physiol. Rev..

[17] Han W., Tellez L.A., Perkins M.H., Perez I.O., Qu T., Ferreira J., Ferreira T.L., Quinn D., Liu Z.W., Gao X.B. (2018). A neural circuit for gut-induced reward. Cell.

[18] Browning K.N., Kalyuzhny A.E., Travagli R.A. (2002). Opioid peptides inhibit excitatory but not inhibitory synaptic transmission in the rat dorsal motor nucleus of the vagus. J. Neurosci..

[19] Travagli R.A., Anselmi L. (2016). Vagal neurocircuitry and its influence on gastric motility. Nat. Rev. Gastroenterol. Hepatol..

[20] Zheng H., Kelly L., Patterson L.M., Berthoud H.R. (1999). Effect of brain stem NMDA-receptor blockade by MK-801 on behavioral and fos responses to vagal satiety signals. Am. J. Physiol. Regul. Integr. Comp. Physiol..

[21] Hanson J.E., Yuan H., Perszyk R.E., Banke T.G., Xing H., Tsai M.C., Menniti F.S., Traynelis S.F. (2024). Therapeutic potential of N-methyl-D-aspartate receptor modulators in psychiatry. Neuropsychopharmacology.

[22] Seeburg P.H. (1993). The TiPS/TINS lecture: The molecular biology of mammalian glutamate receptor channels. Trends Pharmacol. Sci..

[23] Lipton S.A. (2004). Failures and successes of NMDA receptor antagonists: Molecular basis for the use of open-channel blockers like memantine in the treatment of acute and chronic neurologic insults. NeuroRx.

[24] Smith P.F. (2003). Therapeutic N-methyl-D-aspartate receptor antagonists: Will reality meet expectation?. Curr. Opin. Investig. Drugs.

[25] Mony L., Kew J.N., Gunthorpe M.J., Paoletti P. (2009). Allosteric modulators of NR2B-containing NMDA receptors: Molecular mechanisms and therapeutics potential. Br. J. Pharmacol..

[26] Loftis J.M., Janowsky A. (2003). The N-methyl-D-aspartate receptor subunit NR2B: Localization, functional properties, regulation, and clinical implications. Pharmacol. Ther..

[27] Adams R.C., Sedgmond J., Maizey L., Chambers C.D., Lawrence N.S. (2019). Food addiction: Implications for the diagnosis and treatment of overeating. Nutrients.

[28] Volkow N.D., Wang G.J., Tomasi D., Baler R.D. (2013). Obesity and addiction: Neurobiological overlaps. Obes. Rev..

[29] Gearhardt A.N., Corbin W.R., Brownell K.D. (2009). Food addiction: An examination of the diagnostic criteria for dependence. J. Addict Med..

[30] Olsen C.M. (2011). Natural rewards, neuroplasticity, and non-drug addictions. Neuropharmacology.

[31] Hoebel B.G., Hernandez L., Schwartz D.H., Mark G.P., Hunter G.A. (1989). Microdialysis studies of brain norepinephrine, serotonin, and dopamine release during ingestive behavior. Theoretical and clinical implications. Ann. N. Y. Acad. Sci..

[32] Browning K.N., Fortna S.R., Hajnal A. (2013). Roux-en-Y gastric bypass reverses the effects of diet-induced obesity to inhibit the responsiveness of central vagal motoneurones. J. Physiol..

[33] Hoebel B.G., Avena N.M., Bocarsly M.E., Rada P. (2009). Natural addiction: A behavioral and circuit model based on sugar addiction in rats. J. Addict Med..

[34] Gambrill A.C., Storey G.P., Barria A. (2011). Dynamic regulation of NMDA receptor transmission. J. Neurophysiol..

[35] Paulino G., Barbier de la Serre C., Knotts T.A., Oort P.J., Newman J.W., Adams S.H., Raybould H.E. (2009). Increased expression of receptors for orexigenic factors in nodose ganglion of diet-induced obese rats. Am. J. Physiol. Endocrinol. Metab..

[36] Leggio L., Farokhnia M., Kenny P.J., Pepino M.Y., Simmons W.K. (2025). Crosstalk between alcohol use disorder and obesity: Two sides of the same coin?. Mol. Psychiatry.

[37] Nagy J. (2004). The NR2B subtype of NMDA receptor: A potential target for the treatment of alcohol dependence. Curr. Drug Targets CNS Neurol. Disord..

[38] Winder D.G., Egli R.E., Schramm N.L., Matthews R.T. (2002). Synaptic plasticity in drug reward circuitry. Curr. Mol. Med..

[39] Pang Z.P., Han W. (2012). Regulation of synaptic functions in central nervous system by endocrine hormones and the maintenance of energy homoeostasis. Biosci. Rep..

[40] Kauer J.A. (2004). Learning mechanisms in addiction: Synaptic plasticity in the ventral tegmental area as a result of exposure to drugs of abuse. Annu Rev. Physiol..

[41] Carpenter-Hyland E.P., Woodward J.J., Chandler L.J. (2004). Chronic ethanol induces synaptic but not extrasynaptic targeting of NMDA receptors. J. Neurosci..

[42] Fadda F., Rossetti Z.L. (1998). Chronic ethanol consumption: From neuroadaptation to neurodegeneration. Prog. Neurobiol..

[43] Hansen K.B., Yi F., Perszyk R.E., Furukawa H., Wollmuth L.P., Gibb A.J., Traynelis S.F. (2018). Structure, function, and allosteric modulation of NMDA receptors. J. Gen. Physiol..

[44] Cornejo-Pareja I., Molina-Vega M., Gómez-Pérez A.M., Damas-Fuentes M., Tinahones F.J. (2021). Factors Related to Weight Loss Maintenance in the Medium-Long Term after Bariatric Surgery: A Review. J. Clin. Med..

[45] Legendre P., Westbrook G.L. (1991). Ifenprodil blocks N-methyl-D-aspartate receptors by a two-component mechanism. Mol. Pharmacol..

[46] Storey G.P., Riquelme R., Barria A. (2025). Activity-dependent internalization of Glun2B-containing NMDARs is required for synaptic incorporation of glun2A and synaptic plasticity. J. Neurosci..

[47] Antos Z., Żukow X., Bursztynowicz L., Jakubów P. (2024). Beyond NMDA receptors: A narrative review of ketamine’s rapid and multifaceted mechanisms in depression treatment. Int. J. Mol. Sci..

[48] Kavalali E.T., Monteggia L.M. (2012). Synaptic mechanisms underlying rapid antidepressant action of ketamine. Am. J. Psychiatry.

[49] Krystal J.H., Sanacora G., Blumberg H., Anand A., Charney D.S., Marek G., Epperson C.N., Goddard A., Mason G.F. (2002). Glutamate and GABA systems as targets for novel antidepressant and mood-stabilizing treatments. Mol. Psychiatry.

[50] Boku S., Nakagawa S., Toda H., Hishimoto A. (2018). Neural basis of major depressive disorder: Beyond monoamine hypothesis. Psychiatry Clin. Neurosci..

[51] Krystal J.H., Kavalali E.T., Monteggia L.M. (2024). Ketamine and rapid antidepressant action: New treatments and novel synaptic signaling mechanisms. Neuropsychopharmacology.

[52] Maier H.B., Pollak C., Moschny N., Toto S., Schlatt C., Eberlein C.K., Sperling W., Kornhuber J., Kahl K.G., Bleich S. (2022). Electroconvulsive therapy and adiposity-related parameters in treatment-resistant depressed patients. J. Neural Transm..

[53] Gomes A.P., Soares A.L.G., Menezes A.M.B., Assunção M.C., Wehrmeister F.C., Howe L.D., Gonçalves H. (2019). Adiposity, depression and anxiety: Interrelationship and possible mediators. Rev. Saude Publica.

[54] Rashidian H., Rosenblat J.D., McIntyre R.S., Mansur R.B. (2020). Leptin, obesity, and response to ketamine. Prog. Neuropsychopharmacol. Biol. Psychiatry.

[55] Vyklick Vyklicky V., Korinek M., Smejkalova T., Balik A., Krausova B., Kaniakova M., Lichnerova K., Cerny J., Krusek J., Dittert I. (2014). Structure, function, and pharmacology of NMDA receptor channels. Physiol. Res..

[56] Wu F.S., Gibbs T.T., Farb D.H. (1991). Pregnenolone sulfate: A positive allosteric modulator at the N-methyl-D-aspartate receptor. Mol. Pharmacol..

[57] Münzberg H., Laque A., Yu S., Rezai-Zadeh K., Berthoud H.R. (2015). Appetite and body weight regulation after bariatric surgery. Obes. Rev..

[58] Strong K.L., Jing Y., Prosser A.R., Traynelis S.F., Liotta D.C. (2014). NMDA receptor modulators: An updated patent review (2013–2014). Expert Opin. Ther. Pat..

[59] Kavalali E.T., Monteggia L.M. (2023). Rapid homeostatic plasticity and neuropsychiatric therapeutics. Neuropsychopharmacology.

[60] Autry A.E., Adachi M., Nosyreva E., Na E.S., Los M.F., Cheng P.F., Kavalali E.T., Monteggia L.M. (2011). NMDA receptor blockade at rest triggers rapid behavioural antidepressant responses. Nature.

[61] Nosyreva E., Szabla K., Autry A.E., Ryazanov A.G., Monteggia L.M., Kavalali E.T. (2013). Acute suppression of spontaneous neurotransmission drives synaptic potentiation. J. Neurosci..

[62] Wolf M.E. (2016). Synaptic mechanisms underlying persistent cocaine craving. Nat. Rev. Neurosci..

[63] Duman R.S., Aghajanian G.K., Sanacora G., Krystal J.H. (2016). Synaptic plasticity and depression: New insights from stress and rapid-acting antidepressants. Nat. Med..

[64] Wu Q., Zheng R., Srisai D., McKnight G.S., Palmiter R.D. (2013). NR2B subunit of the NMDA glutamate receptor regulates appetite in the parabrachial nucleus. Proc. Natl. Acad. Sci. USA.

[65] Zhu S., Paoletti P. (2015). Allosteric modulators of NMDA receptors: Multiple sites and mechanisms. Curr. Opin. Pharmacol..

[66] Tumdam R., Hussein Y., Garin-Shkolnik T., Stern S. (2024). NMDA Receptors in Neurodevelopmental Disorders: Pathophysiology and Disease Models. Int. J. Mol. Sci..

